# Control of Alzheimer's Amyloid Beta Toxicity by the High Molecular Weight Immunophilin FKBP52 and Copper Homeostasis in *Drosophila*

**DOI:** 10.1371/journal.pone.0008626

**Published:** 2010-01-13

**Authors:** Reiko Sanokawa-Akakura, Weihuan Cao, Kirsten Allan, Khyati Patel, Anupama Ganesh, Gary Heiman, Richard Burke, Francis W. Kemp, John D. Bogden, James Camakaris, Raymond B. Birge, Mary Konsolaki

**Affiliations:** 1 Department of Biochemistry and Molecular Biology, University of Medicine and Dentistry of New Jersey, Newark, New Jersey, United States of America; 2 Department of Genetics, Rutgers, The State University of New Jersey, Piscataway, New Jersey, United States of America; 3 School of Biological Sciences, Monash University, Clayton, Victoria, Australia; 4 Department of Preventive Medicine and Community Health, University of Medicine and Dentistry of New Jersey, Newark, New Jersey, United States of America; 5 Department of Genetics, The University of Melbourne, Melbourne, Victoria, Australia; Brigham and Women's Hospital/Harvard Medical School, United States of America

## Abstract

FK506 binding proteins (FKBPs), also called immunophilins, are prolyl-isomerases (PPIases) that participate in a wide variety of cellular functions including hormone signaling and protein folding. Recent studies indicate that proteins that contain PPIase activity can also alter the processing of Alzheimer's Amyloid Precursor Protein (APP). Originally identified in hematopoietic cells, FKBP52 is much more abundantly expressed in neurons, including the hippocampus, frontal cortex, and basal ganglia. Given the fact that the high molecular weight immunophilin FKBP52 is highly expressed in CNS regions susceptible to Alzheimer's, we investigated its role in Aβ toxicity. Towards this goal, we generated Aβ transgenic *Drosophila* that harbor gain of function or loss of function mutations of *FKBP52*. *FKBP52* overexpression reduced the toxicity of Aβ and increased lifespan in Aβ flies, whereas loss of function of *FKBP52* exacerbated these Aβ phenotypes. Interestingly, the Aβ pathology was enhanced by mutations in the copper transporters *Atox1*, which interacts with *FKBP52*, and *Ctr1A* and was suppressed in *FKBP52* mutant flies raised on a copper chelator diet. Using mammalian cultures, we show that *FKBP52* (−/−) cells have increased intracellular copper and higher levels of Aβ. This effect is reversed by reconstitution of *FKBP52*. Finally, we also found that FKBP52 formed stable complexes with APP through its FK506 interacting domain. Taken together, these studies identify a novel role for FKBP52 in modulating toxicity of Aβ peptides.

## Introduction

FKBP52 is a high molecular weight FK506-binding immunophilin, possessing peptidyl-prolyl isomerase (PPIase) activity. It was first identified as a component of steroid hormone receptor hetero-complexes [1] and recently shown to regulate the nuclear localization of the glucocorticoid receptor [2]. It is widely expressed in mammalian tissues including the brain [3] and immunophilins FKBP12 and FKBP52 are up-regulated in regenerating neurons suggesting that they may play a protective or regenerative role following injury [4]–[7], [reviewed in 8]. Immunophilins have also been associated with the processing of Alzheimer's Amyloid Precursor Protein (APP), which can be processed in an amyloidogenic or non-amyloidogenic manner. Overexpression of *Pin1*, a member of the parvulin family of immunophilins [9], reduces Aβ and knockout of *Pin1* increases Aβ production in Alzheimer's disease brains, through the isomerization of the cytoplasmic domain of APP at a phosphorylated Thr_668_/Pro motif [10]. In addition, the APP intracellular domain (AICD) interacts with the peptidyl prolyl isomerase domain of the smaller immunophilin FKBP12 [11].

We have previously explored the existence of additional FKBP52-interacting cellular factors in neuronal cells and found that FKBP52 interacts with Atox1, a metallochaperone for copper [12]. In these studies, expression of FKBP52 in mammalian cells caused lower levels of intracellular copper, suggesting that FKBP52 facilitates copper efflux [12]. Metal dys-homeostasis is instrumental in the pathology of Alzheimer's disease and copper interactions with APP and Aβ, both of which contain copper-binding sites, have been widely documented and implicated in the disease [13]–[14; reviewed in 15]. Although the direct interaction of copper and Aβ is believed to be important for the aggregation and toxicity of the peptide, the copper/Aβ interactions in vivo are complex and multifactorial. In particular, disturbances in both the intracellular compartmentalization of copper as well as in its extracellularly released forms may contribute to Aβ production and toxicity [16]. Additionally, the interaction of APP with copper has been shown to alter levels of Aβ in transgenic mice [17]–[18], although it is not clear whether this is a function of reduced Aβ production or enhanced clearance of the peptide.

Given the importance of copper homeostasis in Alzheimer's disease pathology and our findings and published studies that immunophilins may participate in both of these processes, we examined if FKBP52 might affect Alzheimer's-related processes. To test this, we explored the function of FKBP52 using *Drosophila* genetics and found that mutations in *FKBP52* exacerbate Aβ toxicity while transgenic flies that overexpress wild type *FKBP52* decrease Aβ toxicity. The effects on Aβ phenotypes correlated with altered levels of the peptide, suggesting that *FKBP52* may affect Aβ turnover. We also provide genetic and biochemical evidence that these effects of *FKBP52* can be modulated by altering copper homeostasis during development. Finally, we also provide evidence that FKBP52 binds APP in mammalian cells and alters levels of Aβ. Taken together, our data identify a novel role for FKBP52 in Alzheimer's disease, and suggests that this high molecular weight immunophilin acts on multiple aspects of Aβ metabolism and toxicity.

## Methods

### Drosophila Strains, Rearing and Phenotypic Analysis

**Figure 1:**
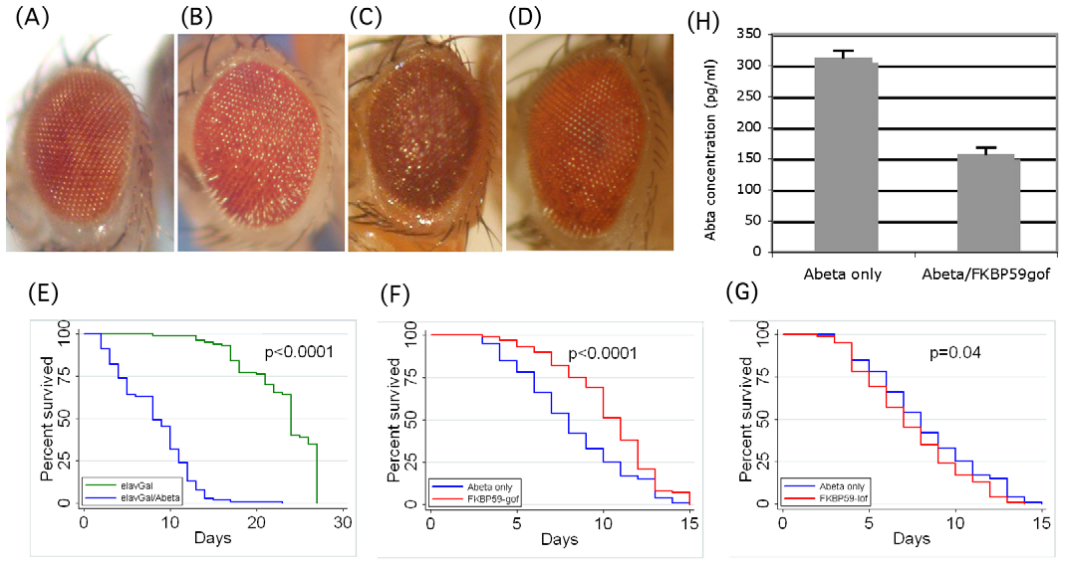
Effects of *dFKBP59* mutations on Aβ42 toxicity in *Drosophila*. (A–D) Eye phenotypes. (A) wild-type eyes of FKBP59^c01413^/FKBP59^c01413^ flies. (B) rough-eye phenotype of *Aβ* flies. (C) enhanced rough-eye phenotype of Aβ flies carrying the loss-of-function mutation *dFKBP59^c01413^*. (D) suppressed rough-eye phenotype of *Aβ* flies carrying the gain-of-function mutation *dFKBP59^EY03538^*. (E–F) Lifespan analysis of Aβ flies carrying *dFKBP59* mutations. (E) CNS-directed expression of Aβ42 (blue) causes a shorter lifespan compared to flies expressing only the elavGal4 driver (green). (F) A gain-of-function mutation in *dFKBP59* rescues the Aβ phenotype. Comparison of *Aβ-only* (blue) and *Aβ/dFKBP59^EY03538^* (red). (G) A loss-of-function mutation in *dFKBP59* rescues the Aβ phenotype. Comparison of *Aβ-only* (blue) and *Aβ/dFKBP59^c01413^* (red). (H) Over-expression of FKBP59 (FKBP59gof) in 15–17 day old Aβ42- expressing flies reduces the levels of total Aβ42.

All flies were kept on yeast-containing media and were raised at 25°C or 29°C. *dFKBP59* and *Atox1* mutations were obtained from the Bloomington Drosophila Stock center (http://flystocks.bio.indiana.edu). *UAS-Ctr1A* flies are described in [19] and do not alter eye morphology when overexpressed (Fig. S1J). Based on information from Flybase [20] and our own analyses, *dFKBP59^c01413^*, *dFKBP59^k00424^* and *dFKBP59^k09010^* are viable loss-of-function mutants and have no effect on the morphology of the eye (Fig. 1A and Fig. S1E-F). Knock-out mutations of the mouse *dFKBP52* gene are viable as well. The fact that three different insertional mutants in *dFKBP59* cause the same phenotype in Aβ flies supports the involvement of this gene in Aβ toxicity. *dFKBP59^EY03538^* causes 2.9-fold up-regulation of the transcript in the presence of Gal4 protein (Fig. S2A) and does not alter eye morphology when overexpressed (Fig. S1K). *Atox1^e01272^*, *Atox1^EY15780^* and *Atox1^f00729^* are also loss of function mutations (Flybase, [20]), which do not affect eye morphology (Fig. S1G–I). Generation of the *GMR-Aβ42* and *UAS-Aβ42* flies is described in [21]. The *UAS-Aβ42* flies used in the lifespan analysis carry additional copies of the transgene, generated by re-mobilization of the original insertion. Additional production of Aβ42 peptides accounts for their dramatically reduced lifespan. Expression of the *Aβ42* transgene in these flies was induced with the *elavGal4* driver strain, which confers pan-neuronal expression [22]. Life-span monitoring was performed as described in [21] and Kaplan-Meier analysis was used to estimate lifespan probability for each genotype and feeding regimen. Time to event was defined as the lifespan for each fly. P-values were calculated using the log rank test on Stata Statistical Software, version 10 (StataCorp. 2007 College Station, TX: StataCorp LP). For the eye phenotype, flies were scored after aging to ∼15–20 days old. The Aβ42 effects are quantified by the severity of the rough eye phenotype, as previously [23]. When modifier mutations are co-expressed in Aβ42-expressing flies, the distribution of phenotypes is shifted to more mild phenotypes in the case of a suppressor, or more severe phenotypes in a case of an enhancer (Fig. S1A–C and Table 1). ∼100 flies were scored per genotype and each experiment was repeated 2 or 3 times and evaluated by a two-tail, two sample-equal variance Student's T-test.

**Table 1:** Effects of dFKBP59 and copper transporter mutations on the Aβ42-induced rough eye phenotype.

		Mutation type	Rough eye phenotype (%±SEM)			Effect
			mild	moderate	severe	
(29°C)***	Aβ only	--	27±0	29.5±1.5	43±1	--
	UAS-Ctr1A	gof *	2.5±2.5 p<0.01	10.2±2.3 p<0.02	87.25±4.8 p<0.01	enhancer
	dFKbp59^EY03538^	gof	54.5±9.6 p<0.1	26±5 p<0.5	19±5 p<0.04	suppressor
(25°C)	Aβonly	--	50±7	47.5±5.5	2.2±1.8	--
	Atox1^e01272^	lof **	15.67±8.95 p<0.07	28±5.1 p<0.08	56.00±9.5 p<0.02	enhancer
	Atox1^EY15780^	lof	12.33±6.9 p<0.03	27.67±1.33 p<0.02	60.33±7.3 p<0.009	enhancer
	Atox1^f00729^	lof	14.5±5.5 p<0.05	57±2 p<0.2	28.5±3.53 p<0.02	enhancer
	dFKbp59^c01413^	lof	11±6 p<0.02	22.7±8.2 p<0.1	66.3±8.77 p<0.01	enhancer
	dFKbp59^k00424^	lof	42.5±9.6 p<0.6	43.5±9.6 p<0.7	13.5±0.5 p<0.02	enhancer
	dFKbp59^k09010^	lof	15.5±15.6 p<0.1	48±6 p<0.9	36.5±9.6 p<0.07	enhancer

*gain-of-function mutation.

**loss-of-function mutation.

***For maximum expression, flies with the UAS/Gal4 system were raised at 29°C. All loss-of-function mutants were raised at 25°C.

### Cu Feeding and Measurements

Males and female flies of the appropriate genotypes were put on food supplemented with CuCl_2_ or the heavy metal chelator, bathocuproinedisulfonic acid (BCS; Sigma-Aldrich, St Louis, MO) as indicated. Progeny grew and were aged on the supplemented food until collected for analysis. Frozen samples of 80–100 isolated 15–17 day old fly heads were digested in 10 ml of 5% nitric acid, with a microwave sample digester CEM Mars 2000 using a modified oyster tissue method. Cu was measured using ICP-MS on a Thermo-elemental X5 instrument. Standard solutions were prepared in 5% HNO_3_. Cu65 was measured and each sample was assayed 3 times per ICP-MS run (variation 5–8%). For the wild-type *oreR* and *Aβ42* flies, the average of 4 independent pools was calculated, showing an average maximum variation around 25–30%, which includes 15% inherent variation of the ICP-MS method. In cultured cells, growth media, and wash media, copper concentrations were determined by electrothermal heated graphite atomizer (HGA) atomic absorption spectrophotometry with a Perkin Elmer Zeeman Model 5100 instrument. Previously described methods [24] were optimized for the instrument, cells, and media specimens. Counted cell populations of 2−9×10^6^ cells were washed with low Cu media and transferred to Eppendorf Safe-Lock (1.5 ml) tubes for digestion. Cells were dried for 1 hour in a heating block at 80°C. The pellet was digested with 100 µL of double-distilled 70% nitric acid (GFS Chemical, Powell, OH), capped and heated overnight at 80°C. The cell digests were diluted with deionized/distilled water prior to analysis. Media was analyzed directly with the addition of an ammonium nitrate modifier to reduce NaCl background interference. A standard reference material that has a known and certified copper concentration (NIST 1571), was purchased from the National Institute for Standards and Technology, and used as quality control specimen. Blanks were also analyzed with each set of media and cell samples. Plasticware used for specimen collection, processing and analysis was acid soaked and rinsed with distilled water prior to use.

### Protein Analysis

Cultured cells were lysed in lysis buffer (20 mM Tris-HCl, 150 mM NaCl, 10 mM Na_4_P_2_O_7_, 2 mM Na_3_VO_4_, 1% Triton X-100, 1 mM PMSF, 20 µg/ml aprotinin). For immunoprecipitation (IP), 0.5∼1.0 mg of total cellular protein was incubated with primary antibody at 4°C overnight, followed by the addition of Protein A/G-Sepharoses and additional incubation at 4°C for 1 h, then resolved by Tris-Tricine gel or SDS-PAGE. Western blotting of cell samples was performed following SDS-PAGE or Tris-Tricine gel electrophoresis and transfer to polyvinylidene difluoride membranes (Milllipore, Billerica, MA). Blots were incubated with primary antibodies, diluted in 5% milk, after which the blots were washed in Tris-buffered saline containing 0.05% Tween 20 and subsequently incubated with horseradish peroxidase-conjugated secondary antibodies. Immunoblots were developed with an enhanced chemiluminescence kit (Western Lightning; PerkinElmer Life Science, Waltham, MA).

### Cell Culture

Human epithelial kidney cells (HEK) were grown in DMEM containing 10% (v/v) FBS at 37°C in 5% CO_2_. Human neuroblastoma cells (SH-SY5Y) were grown in MEM/F12 (1∶1) containing 10%(v/v) FBS, 1 mM sodium pyruvate and nonessential amino acids at 37°C in 5% CO_2_. Wild type and *FKBP52* knockout mouse embryonic fibroblast cells (MEF) were kindly provided by Dr. David Smith (Mayo Clinic, Scottsdale, Arizona) and were grown in MEM containing 10% (v/v) FBS at 37°C in 5% CO_2_.

### Plasmids

Full-length human *FKBP52*, *pCxneo-FKBP52-V5*, was generated as described [12]. Full-length human *APP*, *pCEP4-APP695*, containing myc-tag and flag-tag, was a generous gift from Dr. G. Multhaup (University of Heidelberg) and *pcDNA-APP695* containing flag tag was a generous gift from Dr. T. Mizushima (Kumamoto University). *FKBP52* domain I-II fragment (amino acids 1-261) was subcloned by digested *pCxneo-FKBP52 –V5* with EcoRI into *pTracer-EF/V5-His* for mammalian expression.

### Antibodies

Polyclonal anti-FKBP52 antibody was purchased from Biomol (Plymouth Meeting, PA) and monoclonal anti-FKBP52 antibody was purchased from Stressgen (Ann Arbor, MI). Polyclonal anti-APP antibody (A8717) and monoclonal anti-β-amyloid (6E10) antibody were purchased from Sigma-Aldrich (St Louis, MO). Monoclonal anti-FLAG tag antibody (M2), monoclonal anti-V5 tag antibody and monoclonal anti-Myc tag (9E10) were purchased from Sigma-Aldrich (St Louis, MO), Invitrogen (Carlsbad, CA) and Santa Cruz Biotechnology (Santa Cruz, CA).

### Transient Transfection and Stable Expression

All cell lines were transiently transfected using Lipofectamine 2000 (Invitrogen, Carlsbad, CA). To generate the stable expressing human *APP695* in HEK cells, we transfected with *pcDNA-APP695*, selected by immunoblotting and maintained positive clones in the presence of 200 mg/ml G418 (Gibco- Invitrogen, Carlsbad, CA). To generate the stable expressing human *APP695* in *FKBP52* knockout mouse embryonic fibroblast (MEF) cells, we co-transfected with *pcDNA-APP695* and *pBabe* vector, selected by immunoblotting and maintained positive clones in the presence of 3 mg/ml puromycin (Invitrogen, Carlsbad, CA).

### ELISA for Aβ Peptides

Levels of Aβ peptides in Drosophila heads shown in Fig. 1H were analyzed as described in [21]. For the analysis of Aβ peptides in mammalian cells, stable *APP695-HEK* cells or stable *APP695-FKBP52* knockout MEF cells were transiently transfected with vector or *FKBP52-V5* plasmid for 48 hrs. The medium was changed and the conditioned medium was collected. Medium was centrifuged for 10 min at 13000 rpm to remove cellular debris and adjusted to 25 mM Tris-HCl (pH 7.5) containing 1 mM PMSF, 20 mg/ml aprotinin and 0.5% TritonX100. To measure concentration of Aβ40 or Aβ42, medium was analyzed using an ELISA kit (Covance Research Products Inc. Dedham, MA).

## Results

### *FKBP52* Modifies the Toxic Effects and Levels of Aβ Peptides in *Drosophila melanogaster*

We first investigated the effect of *FKBP52* on the toxicity of Aβ peptides, by a functional genetic approach. We have previously engineered transgenic *Drosophila melanogaster* strains that express human Aβ42 peptides and shown that Aβ expression promotes degeneration of the nervous system with concomitant learning and memory defects, in a dose dependent manner [22], [25]. When Aβ is expressed using an eye-specific promoter, it induces a rough eye phenotype characteristic of inappropriate organization of ommatidia (Fig. 1A–B), whereas expression in the CNS causes reduced lifespan (Fig. 1E). These phenotypes are caused by progressive degeneration of the eye and brain tissue, respectively. We performed genetic interaction analysis in these Aβ-expressing flies by analyzing the modification of Aβ-induced phenotypes in the presence of *FKBP52* mutations. *Drosophila* encodes four known members of the *FKBP* family [20]. Two of those are homologs of the smaller form (*FKBP12*, *FKBP13*), one resembles the atypical form *FKBP39* and the fourth member, *dFKBP59* [26], is a homolog of *FKBP52*, containing three FK506 binding domains and three tetratricopeptide repeats (TPR).

Shown in Table 1 are *Drosophila* strains that carry mutations caused by transposable element insertions in the *Drosophila dFKBP59* gene. We generated flies co-expressing Aβ42 with each of these *dFKBP59* mutations and quantified their Aβ42-induced rough eye phenotype (as described in Methods and Fig. 1SA–C). We found that the loss-of-function mutation *dFKBP59^c01413^* caused enhancement of this phenotype (Fig. 1C), whereas the mutant *dFKBP59^EY03538^*, which over-expresses *dFKBP59*, suppressed the Aβ42-induced rough eye phenotype (Fig. 1D). Two additional loss of function *dFKBP59* mutations were examined, one causing a strong enhancement (*dFKBP59^k00424^*) and the second causing a marginal enhancement (*dFKBP59^k09010^*) of Aβ toxicity (Table 1). All loss of function alleles of *dFKBP59* have wild-type eyes (Fig. 1A, Fig. S1E–F). To further examine this effect, we also tested if *dFKBP59* mutations might alter the lifespan of Aβ42-expressing flies. Figures 1F-G show a comparison between Aβ42-expressing flies with or without a mutation in *dFKBP59*. In the presence of the gain-of-function mutation *dFKBP59^EY03538^*, the lifespan of Aβ42-expressing flies was extended significantly (Fig. 1F; P = 0.0001), suggesting that the *dFKBP59* mutation had a beneficial effect on Aβ42 toxicity. We also tested the loss-of-function mutation *dFKBP59^c01413^* in the same assay and found that it caused the opposite effect (Fig. 1G; P = 0.04), although this was milder than the effect of the gain of function mutation. This could be due to different expressivity of the mutations.

Our results indicate that *FKBP52/dFKBP59* may function independently of its role as a prolyl-isomerase to modify Aβ toxicity, as Aβ does not contain proline residues. Alternatively, dFKBP59 might indirectly affect Aβ phenotypes. In order to further analyze the *dFKBP659* effects on Aβ42, we measured levels of Aβ42 peptides in Drosophila heads over-expressing Aβ and dFKBP59. As shown in Fig. 1H, these flies (Abeta/FKBP59gof) have significantly lower steady state levels of Aβ peptides, consistent with their suppressed eye and lifespan phenotypes. The results of our genetic analysis suggest that *dFKBP59* can modify the toxic phenotypes of Aβ42 peptides by affecting the levels of the peptide.

### Copper Homeostasis Is Linked to Toxicity of Aβ42 in Flies

Our previous studies indicated a role for FKBP52 in the regulation of intracellular copper metabolism [12]. Since altered copper homeostasis has clearly been shown to have a role in Alzheimer's disease [reviewed in 15], we examined whether it might impinge on the toxicity of Aβ in our model system. For this, we manipulated copper levels in Drosophila using two independent approaches and examined effects on Aβ42-induced phenotypes. In the first approach, we increased or decreased available copper by either raising flies in food supplemented with copper or the metal chelator BCS, respectively. In the second approach, we examined toxicity of Aβ in genetic backgrounds where copper transporter genes were mutated.

**Figure 2:**
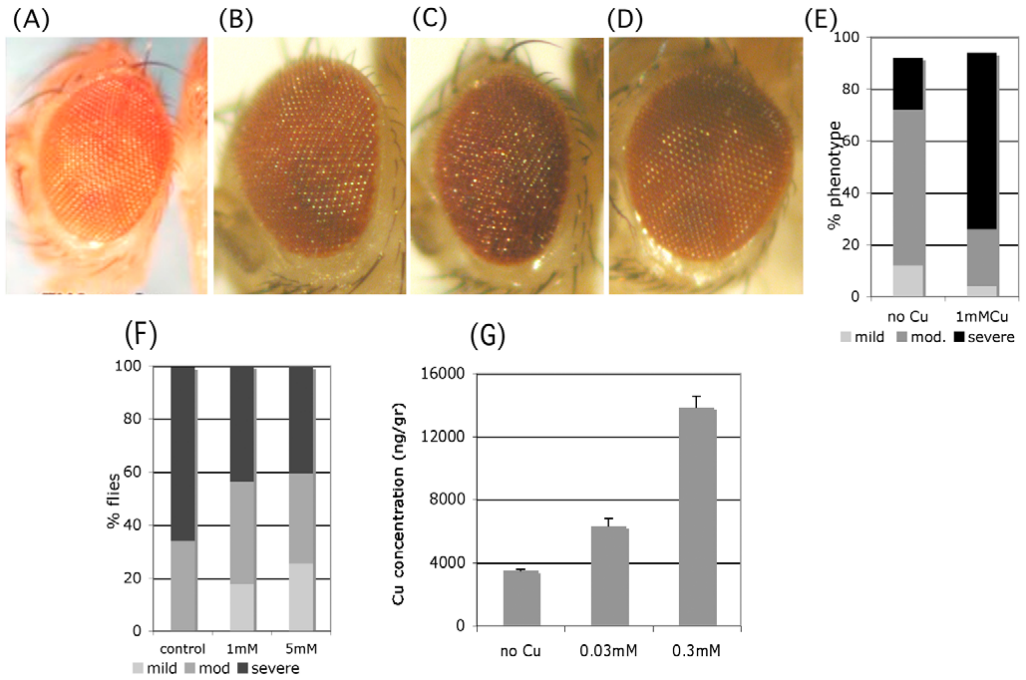
Effects of copper on Aβ42 phenotypes. (A–D) Eye phenotypes of 15–17 day old flies. (A) wild type flies on 1 mM Cu (B) *Aβ* flies on normal food. (C) *Aβ* flies on food supplemented with 1 mM Cu. (D) *Aβ* flies on food supplemented with 1 mM BCS. (E–F) Quantification of the effects of Cu (E) and BCS (F) feeding on the rough eye phenotype. Phenotypes were evaluated as mild (light gray), moderate (dark gray) or severe (black) and the percent distribution of these phenotypes is shown. The graphs show a shift in the distribution of phenotypes when flies are raised on supplemented food. (G) Dose-dependent increase in levels of copper in flies raised on Cu-supplemented food.

Copper-supplemented food does not affect the morphology of the eyes of wild type flies (Fig. 2A). However, Aβ42 flies raised on 1 mM copper had more severe rough eye phenotypes (Fig. 2C) than Aβ42 flies raised on normal food (Fig. 2B). Fig. 2E shows that ∼70% of flies fed 1 mM copper had severe rough eyes, whereas only ∼20% of flies raised on normal food had severe rough eyes, suggesting that copper increased the toxicity of Aβ42 peptides. In order to reduce the amount of available copper, we raised flies on food supplemented with the chelator BCS. Since BCS shows minimal toxicity when fed to flies (data not shown), we fed 1 mM and 5 mM BCS to freshly eclosed flies with eye-specific Aβ42 expression. We examined flies aged to 25 days old and found that BCS feeding ameliorated the Aβ-induced rough eye phenotype (Fig. 2D). Control flies raised on normal food had only moderate and severe eye phenotypes whereas flies fed 1 mM or 5 mM BCS also had mild phenotypes (18% and 25% of the progeny, respectively; Fig. 2F). These experiments demonstrate that altered Cu levels can directly alter Aβ toxicity in flies.

In order to quantify the increase of Cu levels in flies raised on copper-supplemented food, we used ICP-MS to measure copper in fly heads. As shown in Fig. 2G, supplementing the fly food with 0.03 mM or 0.3 mM of copper causes an increase in the steady state levels of copper in *Drosophila* heads, in a dose dependent manner (6,340 ng/g in 0.03CumM and 13,860 ng/g in 0.3 mM copper, as compared to 3,500 ng/g in flies fed normal food). Expression of Aβ42 does not affect the steady state levels of copper in fly heads, which contain less than one third of copper compared to the rest of the body (Fig. S2B). More than 75% of the metal is found in the insoluble fraction of head extracts (Fig. S2C).

### Mutations in the Copper Transporters Atox1 and Ctr1A Modify Aβ42-Induced Rough Eye Phenotypes and Alter Levels of Copper

In order to further analyze the interaction of copper with Aβ, we tested the effects of the Cu transporter genes *Ctr1A* and *Atox1* on Aβ phenotypes. Flies express three isoforms of *Copper transporter 1* [<italic>Ctr1</italic>]; [27; reviewed in 28], which is a transmembrane protein responsible for import of copper ions into the cell [reviewed in 29]. In addition, *Drosophila* carries a homolog of *Atox1* [20], which is a cytoplasmic chaperone responsible for delivery of copper to copper transporting ATPases and hence to the secretory pathway.

**Figure 3:**
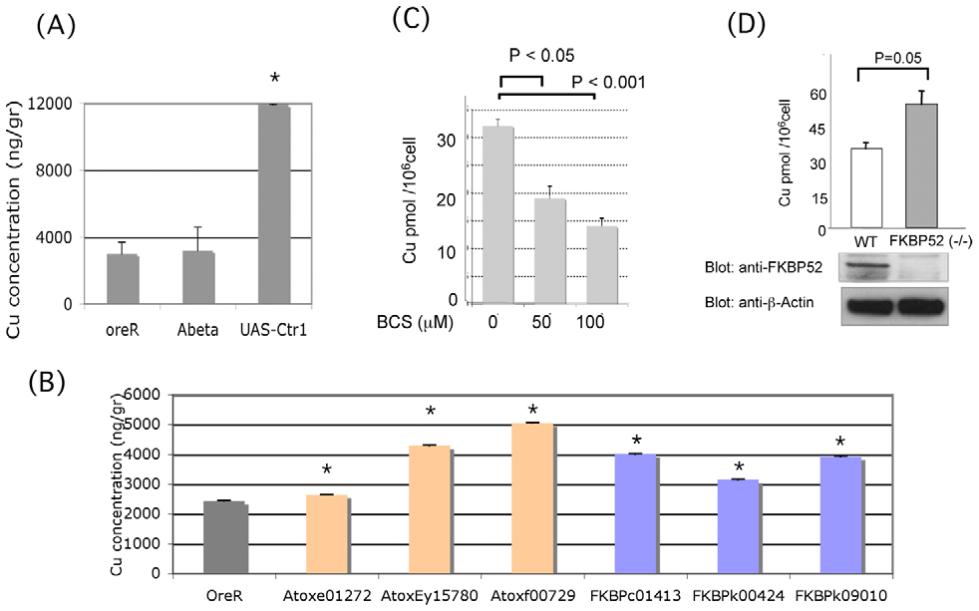
Mutations in *Ctr1*, *Atox1* and *dFKBP59* alter levels of Cu in Drosophila heads and mouse cells. (A–B) Copper measurements in Drosophila heads. (A) Control flies (oreR), flies expressing Aβ42 (Abeta) or flies over-expressing *Ctr1A* (UAS-Ctr1). (B) Wild type flies (oreR) and flies carrying loss-of-function mutations in the *Atox1* and *FKBP59* genes. Stars denote statistical significance. (C–D) Copper measurements in MEF cells. (C) intracellular copper in cells treated with BCS. (D) Intracellular copper in *FKBP52* knock-out MEF cells.

We first examined the rough-eye phenotype of *Drosophila* over-expressing *Ctr1A* and Aβ42 and found that it was enhanced, compared to flies expressing only Aβ42 (Table 1). Given the role of *Ctr1A* in importing Cu into the cell, we hypothesized that the *Ctr1A* effects might be mediated by higher levels of available copper. To test this, we measured the amount of copper in heads of flies over-expressing the copper transporters *Ctr1A* under the control of the *UAS/Gal4* system [30] and found a 4-fold increase in the levels of copper, as compared to control heads (Fig. 3A). The fact that over-expression of *Ctr1A* caused elevated levels of copper supports our hypothesis that the enhancement of the Aβ42 phenotype when this transporter is over-expressed may be due to the presence of elevated copper levels in the cell.

We then examined how mutations in *Atox1* are affecting the Aβ42 phenotype. Table 1 shows that three different loss-of-function alleles of the *Atox1* gene, *Atox1^e01272^*, *Atox1^EY15780^*, and *Atox1^f00729^* cause enhancement of the Aβ42 rough eye phenotype. Atox1 loss of function mutations do not affect eye morphology (Fig. S1G–I). Since Atox1 is involved in the delivery of copper to copper-transporting ATPases in the trans Golgi network (TGN), perturbations in its expression would disturb the proper localization of copper in the cell, potentially exacerbating Aβ toxicity. Supporting this, we found that *Atox1* loss-of-function mutants had more copper than control flies (Fig. 3B). Since we have previously shown that *dFKBP59* interacts with Atox1 and is involved in copper efflux [12], we tested levels of copper in loss of function *dFKBP59* mutant Drosophila heads and found that they had increased levels of copper (Fig. 3B). Although all loss of function mutations of *Atox1* and *dFKBP59* that we examined increased levels of copper, the increases were of varying degrees, perhaps reflecting genetic background effects. It is worth noting however, that the three mutants with the strongest phenotypic effects on Aβ42, *Atox1^EY15780^*, *Atox1^f00729^* and *dFKBP59^c01413^*, showed the higher increases in levels of copper.

We showed the same effects of loss of *FKBP52* function on copper levels, in mammalian cells. Changes in copper levels can be measured in MEF cells treated with 50 mM or 100 mM of the chelator BCS, as shown in Fig. 3C. Using this assay, we showed that immortalized MEF cells obtained from *FKBP52* null mice [*FKBP52*(−/−)] had elevated copper compared to wild type MEF cells (48 pmol/10^6^ cells versus 31 pmol/10^6^ cells; p = 0.05; Fig. 3D), confirming our previous observations that over-expression of FKBP52 causes lower levels of copper [12]. These experiments further support the involvement of *FKBP52* in copper homeostasis and suggest that changes in levels of cellular copper by mutations in *FKBP52* may potentiate the toxic effects of Aβ.

### Synergy between Immunophilin *FKBP52* and Copper Homeostasis Moderates Aβ42 Toxicity

We next performed a genetic interaction analysis, to test whether the effect of *dFKBP59* on Aβ toxicity might be dependent on copper homeostasis. If this were true, we should be able to detect an epistatic relationship between available copper levels and functions of *dFKBP59*, using as a read-out the Aβ42-induced phenotypes in flies. We chose to manipulate the levels of copper by raising *Drosophila* on BCS-supplemented food, a treatment that, as we showed above, improves Aβ42 phenotypes. Our experimental progeny was expressing Aβ42 in the presence of either over-expression or loss-of-function mutations of *dFKBP59* and was tested for effects on the lifespan phenotype, which provides a measure of CNS function.

**Figure 4:**
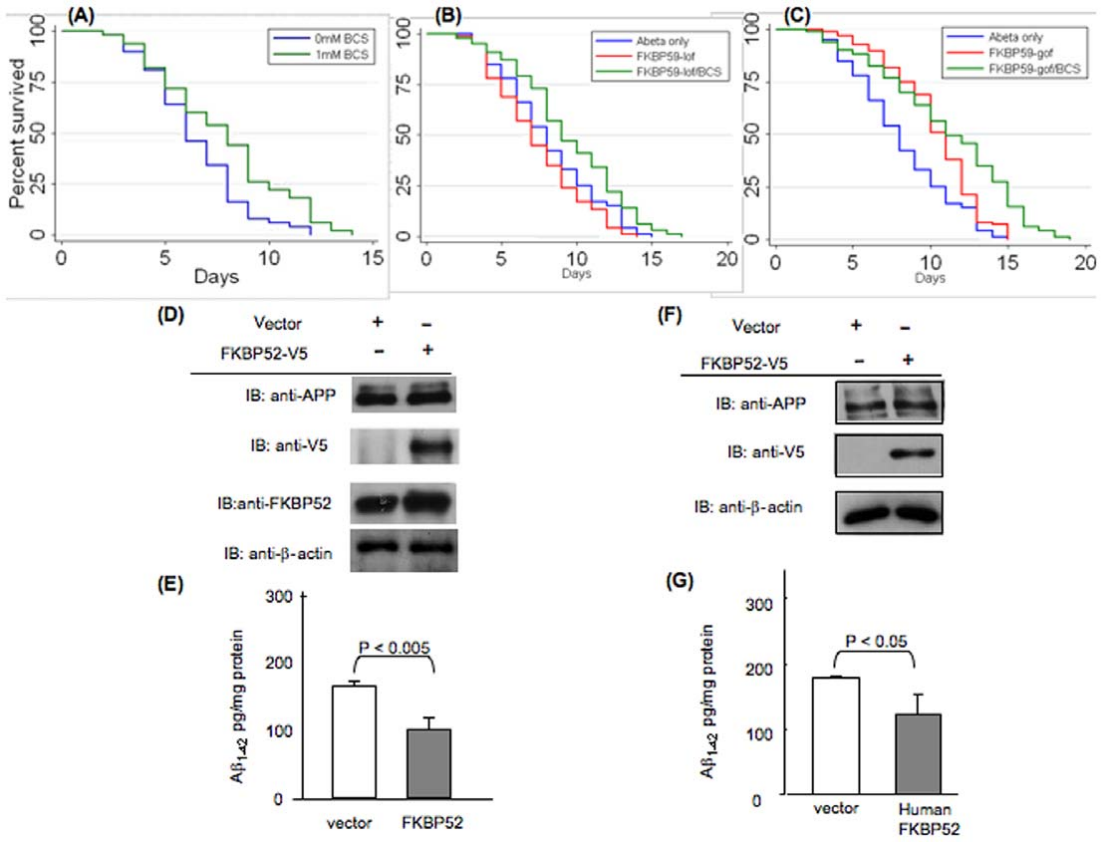
Interaction of dFKBP59 and copper and effects on Aβ levels. (A–C) Lifespan analysis of *Aβ* flies carrying mutations in *dFKBP59* and raised on normal food or food supplemented with 1 mM BCS. (A) *Aβ/BCS* flies (green) compared to *Aβ* flies (blue). (B) *Aβ/dFKBP59^lof^* flies (red) compared to *Aβ/dFKBP59^lof^/BCS* flies (green). (C) *Aβ/dFKBP59^gof^* flies (red) compared to *Aβ/dFKBP59^gof^/BCS* flies (green). (D) APP and FKBP52 expression in APP695-HEK cells transiently transfected with FKBP52. (E) Aβ levels in conditioned medium collected after 24 h from cells in (D). (F) APP and FKBP52 expression in APP695-FKBP52 knockout MEF cells transiently transfected with FKBP52. (G) Aβ levels in conditioned medium collected after 48 h from cells in (F).

As a control, we treated Aβ-expressing flies with BCS and observed a beneficial effect on their lifespan (Fig. 4A). We next treated Aβ flies carrying a loss-of-function mutation of *dFKBP59*, which showed a highly significant increase in lifespan, relative to non-BCS treated flies (Fig. 4B; P = 0.0001). Similarly, treated Aβ flies over-expressing *dFKBP59* showed significantly increased lifespan versus non-treated flies (Fig. 4C; P = 0.0004). The fact that this increase in lifespan is more evident towards the later stages of life may indicate that the effects are dependent on specific levels and/or nature of Aβ oligomeric species. These results suggest that levels of intracellular copper influence the effects of *dFKBP59* on Aβ42 toxicity.

### Role of *FKBP52* in Aβ Metabolism in Mammalian Cells

We next investigated whether FKBP52 affected Aβ levels in mammalian cells expressing APP. Previous studies have suggested a role for other members of the prolyl-isomerase family, such as FKBP12 and Pin1, in promoting the non-amyloidogenic processing of APP [reviewed in 31]. However, no previous study has addressed the involvement of the larger immunophilin FKBP52 in this pathway.

To test the effect of *FKBP52*, we transiently transfected *FKBP52* in HEK cells that had stable expression of *APP695* (Fig. 4D) and assayed levels of Aβ in the conditioned media with an ELISA assay. Fig. 4E shows that overexpression of *FKBP52* reduced the levels of Aβ42 peptides in stable *APP695-HEK* cells. We also transfected human *FKBP52* in *FKBP52*-knockout *APP695-MEF* cells (Fig. 4F) and found that in these cells, Aβ42 levels were also less than those in *FKBP52* knockout cells not transfected with human *FKBP52* (Fig. 4G). The fact that HEK cells with wild-type *FKBP52* and MEF cells with *FKBP52* knockout produce the same amount of Aβ may be due to different steady state levels of Aβ in HEK versus MEF cells. These results suggest that, as in Drosophila, FKBP52 reduces levels of Aβ in mammalian cells as well.

### FKBP52 Interacts with APP

**Figure 5:**
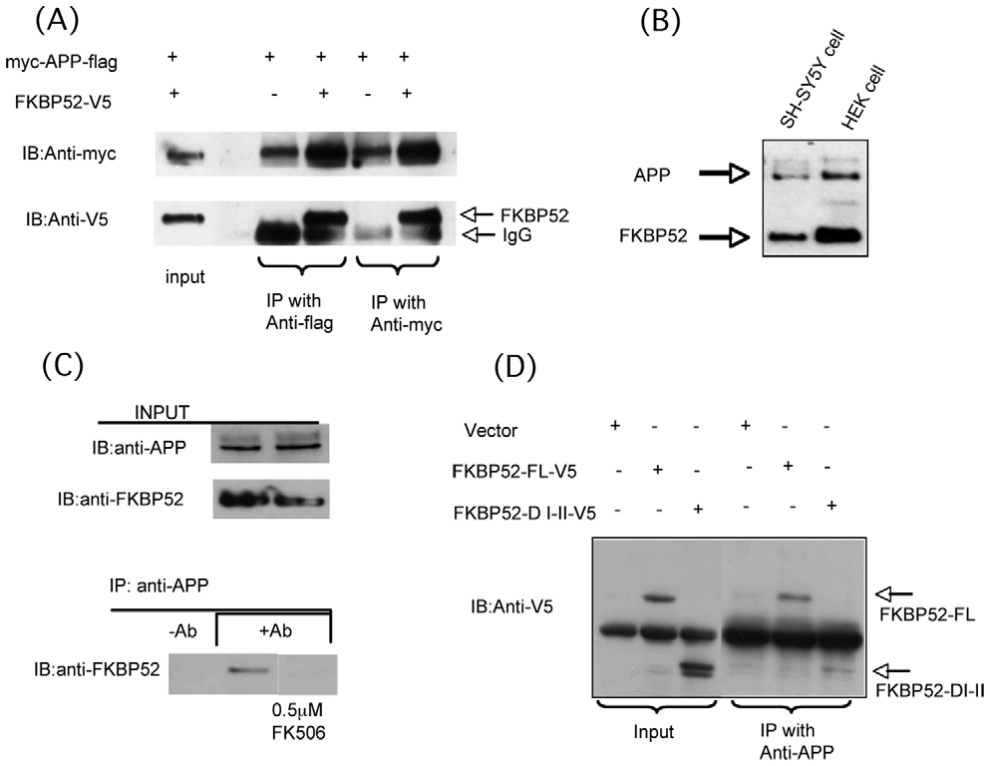
FKBP52 interacts with APP in endogenous and overexpression systems. (A) HEK cells were transiently co-transfected with *Myc-APP-FLAG* and *FKBP52-V5*, immuniprecipitated with anti-Myc or anti-FLAG antibody and detected by western blot using anti-V5 antibody or anti-Myc antibody. (B) Western blot analysis for endogenous expression levels of both APP and FKBP52 in whole-cell lysates from human neuroblastoma cell (SH-SY5Y) and Human Kidney Epithelial cell (HEK). (C) HEK cells were treated with or without 0.5 µM FK506 for 5 hr and immunoprecipitated with anti-APP antibody followed by western blot using anti FKBP52 antibody. Expression level of APP or FKBP52 was detected by western blot using anti-APP or anti-FKBP52 antibody. (D) HEK cells were transiently transfected with full-length FKBP52-V5 or FKBP52 domain I-II-V5 and immunoprecipitated with anti-APP antibody followed by western blot using an anti V5 antibody.

We subsequently investigated whether FKBP52 and APP interact physically. We co-transfected *Myc-APP695-FLAG* and *FKBP52-V5* into HEK cells. As is seen in Fig. 5A, using immunoprecipitation with appropriate antibodies, we were able to detect an interaction between the two proteins. We confirmed this interaction by also testing the pools of endogenous proteins in these cells. As HEK cells express both endogenous *APP* and *FKBP52*, they are suitable for testing the in vivo interaction of APP and FKBP52 (Fig. 5B). Using co-immunoprecipitation, we detected evidence for an interaction between endogenous APP and FKBP52, in cell lysates of HEK cells (Fig. 5C), confirming the results that we obtained with the transfected forms of the two proteins. This interaction was blocked after treatment of the cells with 0.5 mM FK506 (Fig. 5C). Thus, similar to the smaller immunophilin, FKBP12, the larger protein FKBP52 is interacting with APP.

We next sought to determine which domain of FKBP52 is responsible for the interaction with APP. Guided by the interaction of the PPIase domain of FKBP12 with APP, we cloned and transfected the truncated domain I-II of FKBP52, which encompasses amino-acids 1–261 and contains a PPIase domain. As shown in Fig. 5D, the truncated domain of FKBP52 bound APP in a similar fashion to the binding of the full length protein, suggesting that the interaction of FKBP52 with APP is mediated by the PPIase domain. Domain I of FKBP52 associates with various cellular factors, including the glucocorticoid receptor [1], [32] dynein [33], the transient receptor potential channel [TRP; 34], and Atox1 [12]. With the exception of dynein, most of these factors dissociate from FKBP52 after addition of FK506. Since we showed that FK506 can promote dissociation of the endogenous APP/FKBP52 complex (Fig. 5C), we suggest that the PPIase domain plays a direct role in the FKBP52/APP interaction.

## Discussion

The high molecular weight immunophilin FKBP52 belongs to a family of versatile multi-domain proteins that are abundantly expressed in the nervous system and often show increased expression in damaged or degenerating brain regions. In the present study, we have identified a novel function of FKBP52 in Aβ-mediated toxicity using a genetic model in *Drosophila* that expresses Aβ42 peptides. The protective effect of FKBP52 on Aβ toxicity during *Drosophila* aging was evident from the observations that *FKBP52* loss of function mutations potentiated Aβ toxicity, while over-expression of *FKBP52* delayed or suppressed Aβ-induced phenotypes. Moreover, through genetic interactions and chemical approaches, we also found evidence that the effects of FKBP52 may be modulated by changes in intracellular copper homeostasis. These observations are consistent with our previous reports that FKBP52 interacts with the copper efflux machinery, and as such, establish a new aspect of involvement of the immunophilin family in Alzheimer's-related mechanisms. Several lines of evidence link the protective effects of FKBP52 with intracellular copper homeostasis. First, FKBP52 directly interacts with the copper metallochaperone Atox1 [12], a protein that delivers copper to the copper transporting ATPAses ATP7A and ATP7B [reviewed in 35]. Second, through genetic screens in this study, we found that mutations in the copper transport genes *Ctr1A* and *Atox1*, which directly regulate intracellular copper levels, modify Aβ-induced phenotypes in *Drosophila*. Third, chemical manipulation of dietary copper levels also decreases or increases, respectively, the protective effect of FKBP52 on Aβ toxicity. Finally, MEF cells isolated from *FKBP52*(−/−) mice show increased levels of copper compared to wild type MEF cells and over-expression of FKBP52 causes efflux of copper [12].

The metallobiology of copper plays a significant role in several neurodegenerative conditions. Interestingly, copper influences the aggregation properties of “toxic peptides” that contribute to these conditions, including beta amyloid, prion protein and α-synuclein as all three of these agents can bind copper ions [reviewed in 36]. The presence of copper in mildly acidic conditions induces aggregation of the Aβ peptide [13] and may exacerbate pathology linked to Aβ deposition. Our experiments show that all genetic manipulations that increased levels of copper, also enhanced the Aβ phenotypes. Copper manipulations were mediated either by increased copper entry, through over-expression of the plasma membrane transporter *Ctr1A*, or by misregulation of cytoplasmic copper trafficking through loss-of-function mutations of the *Atox1* and *FKBP52* genes. The enhancement of Aβ phenotypes by increasing dietary copper also supports these observations. Since copper is delivered to the trans-Golgi network (TGN) by the cytoplasmic transporter Atox1, we hypothesize that the interaction of Aβ with copper may at least partially take place in the TGN. Given the mildly acidic pH of the TGN [37]–[38], increased levels of copper in this compartment would lead to enhancement of Aβ toxicity and result in more severe Aβ-induced phenotypes, possibly through the induction of oxidative stress. Supporting the role of oxidative damage, over-expression of the anti-oxidative stress gene ferritin heavy chain suppressed the Aβ42-induced short lifespan in *Drosophila* [39]. We further found that flies over-expressing *dFKBP59* had lower levels of Aβ peptides, consistent with their suppressed phenotypes. Based on this, we suggest that dFKBP59 over-expression leads to increased Aβ turnover.

The interaction of FKBP52 with the transporter Atox1 presents a novel aspect of copper metabolism. FKBP52 participates in many cellular processes, including the translocation of steroid receptor complexes to the nucleus through interactions with dynein [reviewed in 40]. FKBP52 also has chaperone activity shown by suppression of the aggregation of heat-denatured citrate synthase [41]. We propose that FKBP52 may be required for the proper function of Atox1. Further analysis using double mutants of *Atox1* and *FKBP52* and examining their effects on Aβ toxicity would be needed in order to confirm this hypothesis.

We also examined the effects of FKBP52 in mammalian cells expressing APP. Unlike Aβ, which does not contain proline amino acids and is not regulated by prolyl isomerization, the APP holo-enzyme binds the prolyl-isomerase Pin1 in its intracellular tail [10] and it also interacts with the small immunophilin FKBP12 [11]. In the current studies we provide evidence that APP also binds FKBP52, via its FK506 binding domain and that *FKBP52*(−/−) cells have higher levels of Aβ peptides than the same knock-out cells reconstituted with *FKBP52*. The physical interaction of FKBP52 with APP suggests that this large immunophilin, in addition to altering Aβ levels, may have a role on the metabolism of APP. The effects of smaller members of the immunophilin family on the processing of APP would support such a role and future experimentation will address this hypothesis. In support of this novel role of immunophilins, we showed recently that a mutation in the *Drosophila Ryanodine receptor homolog Rya-r44F* could modify a APP-overexpression associated phenotype [42]. The FKBP12 protein interacts with ryanodine receptors [43], further implicating signaling through the immunophilin famlily with APP metabolism.

In summary, our studies show that the large immunophilin FKBP52 modulates Aβ toxicity, possibly through a mechanism that involves homeostasis of cellular copper. Our data does not rule out the possibility that the effects of *FKBP52* mutations and metal transport act in parallel pathways, however, it provides indirect evidence for a possible mechanistic link between these respective pathways. Examination of effects of Aβ in *FKBP52* knock-out mice will further validate our observations. The function of immunophilins is modulated by the FK506 family of ligands, several members of which have been developed to bind their targets without causing immune suppression. It will be interesting to examine if such ligands can modify the interaction of FKBP52 with APP and Aβ. We have evidence that the FK506-binding domain of FKBP52 is involved in the binding with APP, suggesting that immunophilin ligands may interfere with this interaction. Further studies will show whether these ligands are also involved in modulating toxicity of Aβ and may open the field for the development of a novel class of agents against Alzheimer's disease.

## Supporting Information

Figure S1 Eye phenotypes of Aβ expressing flies and FKBP59 and Atox1 mutants. (A–C) Rough eye phenotype of Aβ42 flies. (A) Mild, (B) Moderate, (C) Severe. (D–I) wild-type eye phenotype. (D) wild-type oreR flies, (E) FKBP59^k09010^/+ flies, (F) FKBP59^k00424^/+ flies, (G) Atox1^e01272^/Atox1^e01272^ flies, (H) Atox1^EY15780^/Atox1^EY15780^ flies, (I) Atox1^f00729^/Atox1^f00729^ flies. (J) eyGal/+; dFKBP59^EY03538^/+ flies, (K) eyGal/+; UAS-Ctr1A flies.
(6.08 MB TIF)

Figure S2 Analysis of dFKBP59 RNA and copper levels. (A) RNA levels in fly heads over-expressing FKBP59^EY03538^ compared to control flies, measured by real-time PCR analysis, as described in [21]. (B) Cu concentration in pools of 100 flies, aged to 15 days. Bodies contain the majority of Cu. (C) Cu concentration in pools of 60–80 flies, aged to 15 days. Control and flies expressing one or two copies of the Ab42 transgene were analyzed. From each sample, the supernatant (containing the soluble fraction) and pellet (containing the insoluble fraction) were analyzed separately. The majority of Cu is found in the insoluble fraction.
(0.79 MB TIF)
