# Supplementary figures and images for: Control of Alzheimer's Amyloid Beta Toxicity by the High Molecular Weight Immunophilin FKBP52 and Copper Homeostasis in Drosophila
Source: PLoS One. 2010 Jan 13;5(1):e8626. doi: 10.1371/journal.pone.0008626 (PMC2801609; doi:10.1371/journal.pone.0008626)

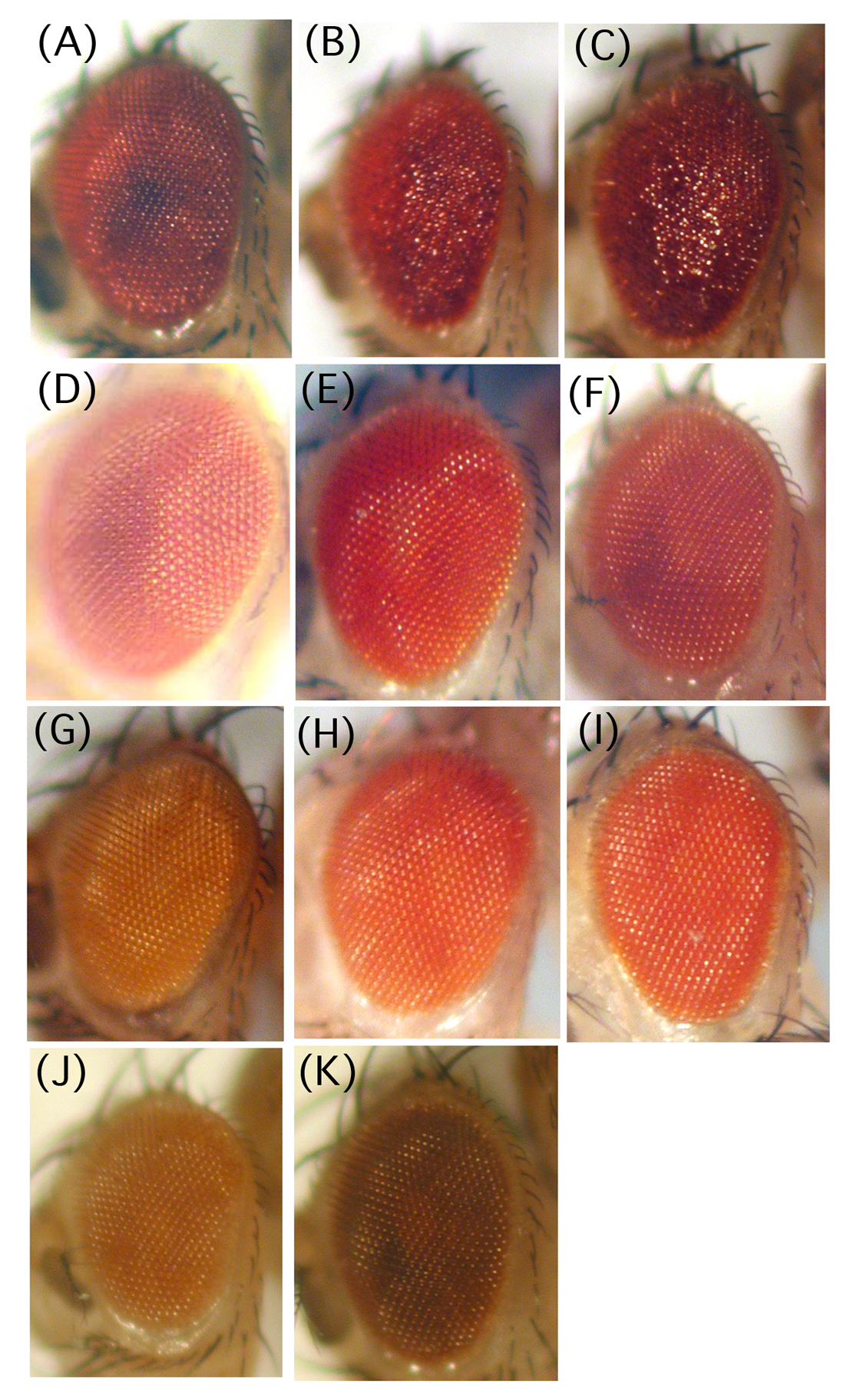

Supplement: Figure S1 — Eye phenotypes of Aβ expressing flies and FKBP59 and Atox1 mutants. (A–C) Rough eye phenotype of Aβ42 flies. (A) Mild, (B) Moderate, (C) Severe. (D–I) wild-type eye phenotype. (D) wild-type oreR flies, (E) FKBP59k09010/+ flies, (F) FKBP59k00424/+ flies, (G) Atox1e01272/Atox1e01272 flies, (H) Atox1EY15780/Atox1EY15780 flies, (I) Atox1f00729/Atox1f00729 flies. (J) eyGal/+; dFKBP59EY03538/+ flies, (K) eyGal/+; UAS-Ctr1A flies. (6.08 MB TIF) [file pone.0008626.s001.tif]

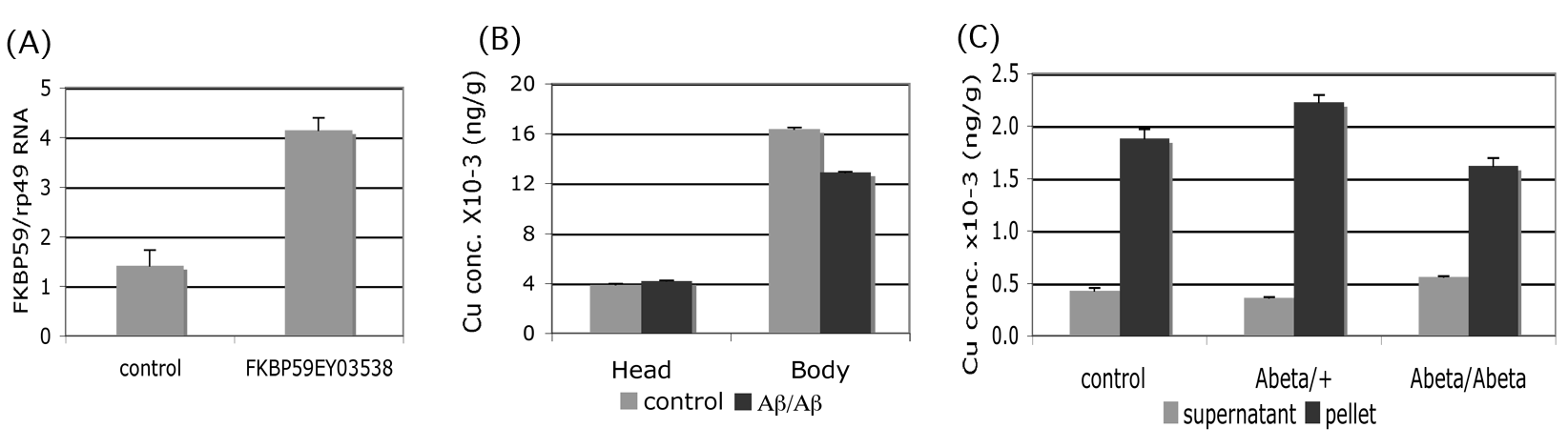

Supplement: Figure S2 — Analysis of dFKBP59 RNA and copper levels. (A) RNA levels in fly heads over-expressing FKBP59EY03538 compared to control flies, measured by real-time PCR analysis, as described in [21]. (B) Cu concentration in pools of 100 flies, aged to 15 days. Bodies contain the majority of Cu. (C) Cu concentration in pools of 60–80 flies, aged to 15 days. Control and flies expressing one or two copies of the Ab42 transgene were analyzed. From each sample, the supernatant (containing the soluble fraction) and pellet (containing the insoluble fraction) were analyzed separately. The majority of Cu is found in the insoluble fraction. (0.79 MB TIF) [file pone.0008626.s002.tif]
